# Correction: Metasurface-enhanced infrared spectroscopy in multiwell format for real-time assaying of live cells

**DOI:** 10.1039/d3lc90071a

**Published:** 2023-07-26

**Authors:** Steven H. Huang, Giovanni Sartorello, Po-Ting Shen, Chengqi Xu, Olivier Elemento, Gennady Shvets

**Affiliations:** a School of Applied and Engineering Physics, Cornell University Ithaca New York 14853 USA hh623@cornell.edu gshvets@cornell.edu; b Caryl and Israel Englander Institute for Precision Medicine, Weill Cornell Medicine New York NY 10021 USA ole2001@med.cornell.edu; c Meinig School of Biomedical Engineering, Cornell University Ithaca NY 14853 USA

## Abstract

Correction for ‘Metasurface-enhanced infrared spectroscopy in multiwell format for real-time assaying of live cells’ by Steven H. Huang *et al.*, *Lab Chip*, 2023, **23**, 2228–2240, https://doi.org/10.1039/d3lc00017f.

The authors regret that funding support from grant UG3CA244697 was mistakenly included in the Acknowledgements section. The correct Acknowledgements section is given below:

 

The research reported here was supported by the National Cancer Institute of the National Institutes of Health under award number R21CA251052 and by the National Institute of General Medical Sciences of the National Institutes of Health under award number R21GM138947. OE is supported by UL1TR002384, R01CA194547, P01CA214274 grants from the National Institutes of Health and LLS SCOR grants 180078-02, 7021-20, 180078-01. This work was performed in part at the Cornell NanoScale Facility, a member of the National Nanotechnology Coordinated Infrastructure (NNCI), which is supported by the National Science Foundation (Grant NNCI-2025233). The authors thank Dr. Ye Fang (Corning Inc.) for insightful discussions on the experimental design using PAR peptides.

 

The Royal Society of Chemistry apologises for these errors and any consequent inconvenience to authors and readers.

## Supplementary Material

